# Revolutionizing *in vivo* therapy with CRISPR/Cas genome editing: breakthroughs, opportunities and challenges

**DOI:** 10.3389/fgeed.2024.1342193

**Published:** 2024-02-01

**Authors:** Arturo Macarrón Palacios, Patrick Korus, Bodo G. C. Wilkens, Najmeh Heshmatpour, Sarita R. Patnaik

**Affiliations:** GenCC GmbH & Co. KG, Heidelberg, Germany

**Keywords:** CRISPR/Cas, genome editing, cancer, personalized therapies, precision medicine, *in vivo* trials

## Abstract

Genome editing using the CRISPR/Cas system has revolutionized the field of genetic engineering, offering unprecedented opportunities for therapeutic applications *in vivo*. Despite the numerous ongoing clinical trials focusing on *ex vivo* genome editing, recent studies emphasize the therapeutic promise of *in vivo* gene editing using CRISPR/Cas technology. However, it is worth noting that the complete attainment of the inherent capabilities of *in vivo* therapy in humans is yet to be accomplished. Before the full realization of *in vivo* therapeutic potential, it is crucial to achieve enhanced specificity in selectively targeting defective cells while minimizing harm to healthy cells. This review examines emerging studies, focusing on CRISPR/Cas-based pre-clinical and clinical trials for innovative therapeutic approaches for a wide range of diseases. Furthermore, we emphasize targeting cancer-specific sequences target in genes associated with tumors, shedding light on the diverse strategies employed in cancer treatment. We highlight the various challenges associated with *in vivo* CRISPR/Cas-based cancer therapy and explore their prospective clinical translatability and the strategies employed to overcome these obstacles.

## 1 Introduction

The initial discovery of CRISPR/Cas (Clustered Regularly Interspaced Short Palindromic Repeats and CRISPR-associated proteins) technology occurred in the early 1990s in the bacterium *Escherichia coli*, a prevalent gut microbe (Ishino et al., 1987; Mojica et al., 1993; Ishino et al., 2018). Subsequently, CRISPR/Cas was discovered as a pivotal component of the prokaryotic adaptive immune system, playing a crucial role in defending against invading genetic elements (Mojica et al., 2000). Despite this early observation, the true potential of CRISPR/Cas as a formidable tool for genome editing was not fully realized until 2012, when Jennifer Doudna and Emmanuelle Charpentier made a seminal discovery (Jinek et al., 2012). They demonstrated that the CRISPR/Cas9 system could be engineered to precisely target and cleave specific DNA sequences within a broad spectrum of organisms, including *Homo sapiens*. This groundbreaking genome editing technology has revolutionized the way scientists study and treat genetic diseases, and since then it has been extensively utilized in both basic and applied research. Industrial biotechnology, agriculture, horticulture, dairy, and poultry sectors have advanced significantly in recent years due to the improvement of CRISPR/Cas genome editing.

CRISPR/Cas9 therapy possesses clear advantages over conventional gene therapies, such as gene replacement therapy, RNA interference (RNAi) therapy, and antisense therapy (Cong et al., 2013; Boettcher and McManus, 2015). CRISPR/Cas9 technology has enabled scientists to make precise changes to the DNA of living organisms, including humans, allowing for the insertion, deletion, or substitution of specific genes. This has been used to correct disease-causing DNA mutations ranging from a single base pair to large deletions, and even the insertion of a missing gene, which can be precisely and permanently integrated into the host genome with just a single genome editing event. This technology offers high accuracy in target gene recognition, minimizing the activation of oncogenes and the risk of mutagenesis. The emergence of CRISPR/Cas9 therapy serves as a powerful reminder that the age of precision medicine is rapidly approaching.

The versatility of CRISPR/Cas9 technology has been demonstrated in both *in vitro* and *in vivo* models, including human cell lines and animal models (Cong et al., 2013). This has allowed researchers to study the effects of gene editing on various diseases and develop new therapies for conditions such as hemophilia, sickle cell anaemia, and cystic fibrosis, to name a few. In addition, the technology has also been used in basic research to study the function of specific genes and understand the underlying mechanisms of various diseases.

Overall, the CRISPR/Cas genome editing technology has dramatically transformed the field of genetics and holds tremendous potential for the future of human disease therapy (Uddin et al., 2020). While there are still challenges that need to be addressed, such as the risk of off-target effects and the ethical and regulatory concerns associated with human gene editing, the future of CRISPR/Cas looks very promising, and it is likely to play an increasingly important role in the development of new treatments for genetic diseases and other medical applications.

This review aims to provide a comprehensive and up-to-date overview of the progress and advancements in the field of *in vivo* therapy using CRISPR/Cas technology. Focusing on the outcomes of clinical trials utilizing CRISPR/Cas, we will cover various diseases with a particular emphasis on cancer therapy. In addition, we will explore the strategies being developed to optimize specificity and minimize off-target effects and will delve into the challenges faced in the implementation of *in vivo* CRISPR/Cas therapy. This review will also provide the latest perspectives on the limitations and opportunities in this rapidly evolving field, with a particular emphasis on the prospects and advancements in CRISPR/Cas genome editing.

## 2 Overview of CRISPR/Cas system

The CRISPR/Cas system is a prokaryotic immune system that provides resistance against invading genetic elements. This immune system is acquired when exogenous DNA is processed by a Cas nuclease into small DNA fragments and incorporated as spacers between conserved repeated sequences within the CRISPR locus of the host genome. These spacers serve as transcriptional templates for processing mature CRISPR RNA (crRNA) molecules, which recognize and guide the Cas nuclease for DNA cleavage of invading viruses and phages. Upon DNA recognition, cleavage occurs through double-strand breaks (DSBs) proximate to the protospacer adjacent motif (PAM) induced by the Cas nuclease (Gasiunas et al., 2012; Jinek et al., 2012).

**FIGURE 1:**
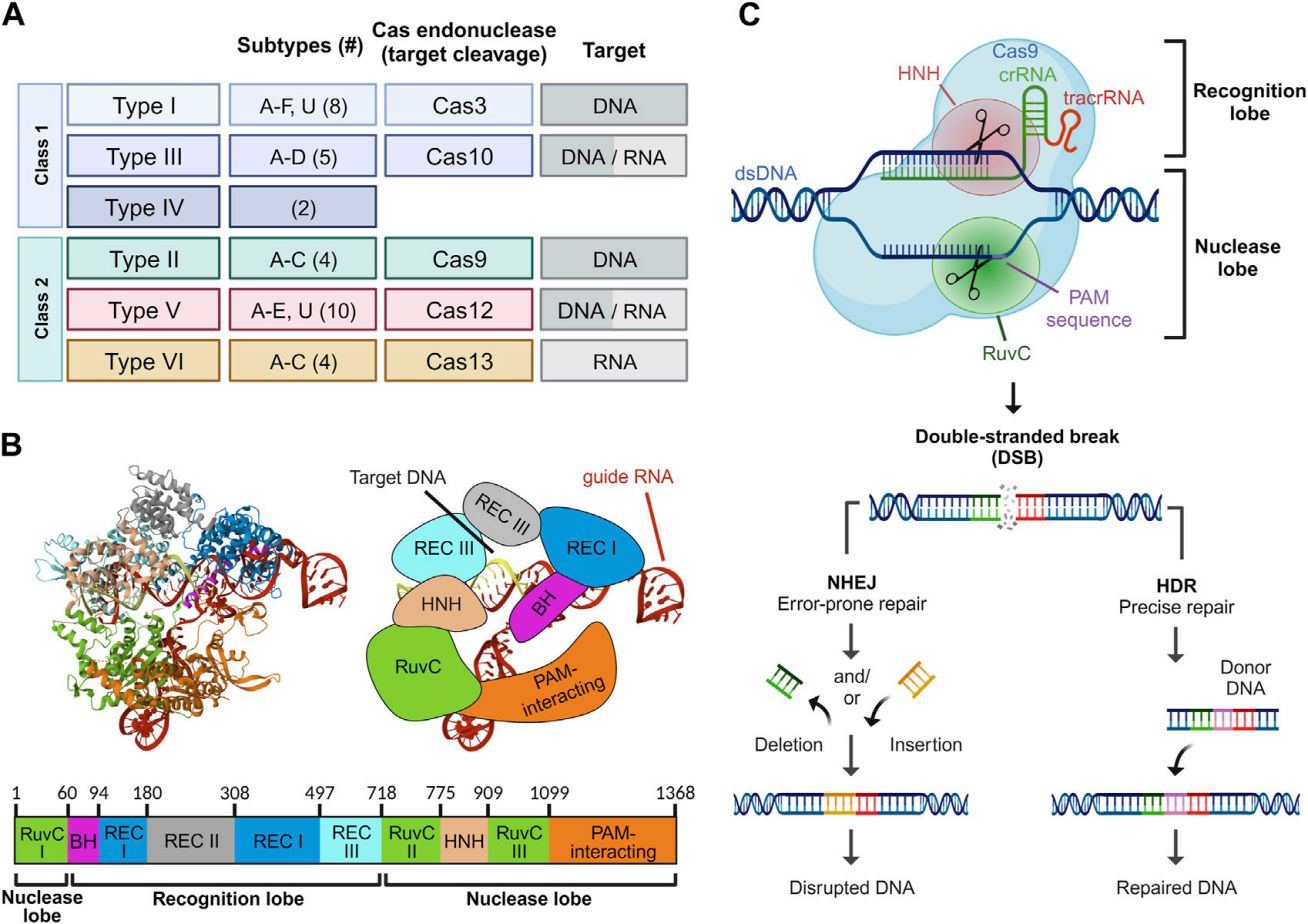
Overview of the CRISPR/Cas platform. Classification of the CRISPR/Cas system based on the class, type, and subtype. The number of subtypes within each CRISPR/Cas type is bracketed. For each type, the Cas endonuclease responsible for target cleavage of DNA and/or RNA is denoted **(A)**. Structure of *Streptococcus pyogenes* Cas9 in complex with guide RNA and target DNA. The protein domains are depicted in crystal (left), schematic (right), and map (bottom) form. Crystal structure was rendered from RCSB PDB ID: 4OO8 (Shin et al., 2017) **(B)**. Mechanism of action of CRIPSR/Cas9. Upon guide RNA-mediated recognition of the PAM sequence, a double-strand break is induced in the target DNA. The DNA damage is subsequently repaired either through error-prone non-homologous end joining (NHEJ) or precise homology-directed repair (HDR) **(C)**.

Based on the structural and functional characteristics of the Cas proteins, the CRISPR/Cas system can be classified into two major classes: Class I (type I, III, and IV) and Class II (type II, V, and VI), and further subdivided into six types and 33 subtypes employing varying sets of Cas proteins and therefore showing characteristic properties (Figure 1A) (Makarova et al., 2015; Mohanraju et al., 2016). Class I systems consist of multi-subunit effector Cas protein complexes, while Class II systems use single Cas protein effectors. Class I systems are complex and diverse defense mechanisms against foreign genetic elements in bacteria. Class I CRISPR systems encompass multiple subtypes, including Type I (I-A, I-B, I-C), Type III (III-A, III-B), and Type IV. Each subtype utilizes distinct multi-subunit complexes and Cas proteins for target recognition, DNA cleavage, and nucleic acid degradation for diverse defense mechanisms against foreign genetic elements in bacteria. Class II systems have garnered particular attention due to their relative simplicity in structure and mechanism of action (Liu et al., 2020). Type II CRISPR/Cas9 is a well-studied and extensively used Cas protein in genetic engineering due to its ease of use and versatility in creating targeted gene modifications (Saijo, 2012). As a result, Cas9 has been extensively utilized for genome editing in diverse organisms, and its potential applications in various fields such as medicine, agriculture, and biotechnology continue to be explored.

The CRISPR/Cas9 system consists of distinct components, each playing a critical role in achieving the desired outcomes. The Cas9 protein is the central component of the CRISPR/Cas9 system and comprises several functional domains essential for its function (Anders et al., 2014). The *N*-terminal region of the protein contains an arginine-rich domain responsible for binding to the negatively charged DNA molecule. This region is followed by the recognition domain, which interacts with the guide RNA (gRNA) to form a complex with the Cas9 protein (Desai et al., 2021). The REC1 and REC2 domains in Cas9 are responsible for binding to the guide RNA and the DNA target, respectively. The HNH and RuvC nuclease domains are present in the C-terminal region of the protein and responsible for the cleavage of the DNA strands at the target site (Figures 1B,C) (Jinek et al., 2012).

The guide RNA in the CRISPR/Cas9 system comprises two distinct RNA segments: the CRISPR RNA (crRNA) and the trans-activating CRISPR RNA (tracrRNA), each playing specific roles in guiding the Cas9 nuclease. The crRNA segment is typically 18–20 nucleotides in length and provides specificity through the so-called spacer sequence, which is complementary to the target DNA sequence. On the other hand, the tracrRNA consists of an 89-nucleotide sequence of loops and serves as a scaffold for the Cas9 nuclease, providing a structure to direct the Cas9 to the target site. Additionally, the Cas9 protein requires a protospacer adjacent motif (PAM) sequence adjacent to the target sequence to initiate cleavage of the DNA (Anders et al., 2014). Single guide RNA (sgRNA) is an engineered RNA molecule composed of a fusion between crRNA and tracrRNA (Jinek et al., 2012). Several attempts have been made to improve the sgRNA structure (Dang et al., 2015; Kim et al., 2020). In a study, Dang *et al.* demonstrated that extending the duplex by four to six nucleotides and introducing a C or G mutation at position 4 within the continuous sequence of Ts, which constitutes the pause signal for RNA polymerase III, leads to a striking optimization of knockout efficiency (Dang et al., 2015). Both gRNA and Cas nuclease form a ribonucleoprotein (RNP) complex. The CRISPR/Cas9 system is a highly complex molecular machinery, requiring precise interactions between its components to achieve accurate genome editing. Optimal design and utilization of these components are vital for effective and efficient genome editing applications.

### 2.1 Mechanisms of CRISPR/Cas9 genome editing

The CRISPR/Cas9 genome editing mechanism typically involves three distinct steps: recognition, cleavage, and repair (Gaj et al., 2013). During the recognition step, the ribonucleoprotein complex, guided by the gRNA, identifies and binds to the specific DNA target sequence. In the cleavage step, the Cas9 protein introduces a double-strand break in the target DNA sequence. This precise gene editing technique has been utilized to knockout specific genes, including oncogenes implicated in cancer therapy development (Zhang et al., 2014; Sánchez-Rivera and Jacks, 2015; Hazafa et al., 2020).

The application of sgRNA in the CRISPR/Cas9 system has revolutionized genome engineering and has enabled researchers to modify and study gene function with unprecedented precision. The resulting DNA damage can be repaired either through non-homologous end joining (NHEJ) or homology-directed repair (HDR) mechanisms, thereby leading to the generation of cells or organisms with specific genetic alterations (Haber, 2000; Shao et al., 2016; Liu et al., 2019). NHEJ is an error-prone repair pathway that directly re-ligates the broken DNA ends, often leading to the insertion or deletion (indel) of nucleotides at the cleavage site (Lieber, 2011; Rodgers and Mcvey, 2016). The consequence of NHEJ repair is the introduction of indels at the cleavage site, which can disrupt gene function by causing frame-shift mutations or premature stop codons (Lieber, 2011; Chang et al., 2017). NHEJ is the predominant DNA repair pathway in mammalian cells and can occur throughout the cell cycle. In contrast, HDR is restricted to the late S and G2 phases and relies on a homologous DNA template to repair the double-strand break with high fidelity (Haber, 2000; Smirnikhina et al., 2022). This template can be a synthetic oligonucleotide, a plasmid, or a chromosome that shares homology with the genomic region flanking the cleavage site. HDR can be used to introduce precise alterations, such as point mutations or insertions, or to replace a mutated gene with a corrected version (Wang B. et al., 2015). HDR-mediated repair can result in precise correction of genetic mutations or targeted modification of gene expression. This has been proven to show significant therapeutic implications for diseases caused by a single gene mutation or aberrant gene regulation, such as inherited blood disorders or genetic blindness (Maeder et al., 2019; Brusson and Miccio, 2021; Frangoul et al., 2021). Moreover, the use of CRISPR/Cas9 in conjunction with HDR-mediated repair allows for the generation of genetically modified organisms with specific alterations or knock-ins. However, the efficiency of HDR repair can vary depending on the delivery method and the quality of the homologous template (Figure 1C) (Liang et al., 1998).

### 2.2 CRISPR/Cas toolkit expansion

**FIGURE 2:**
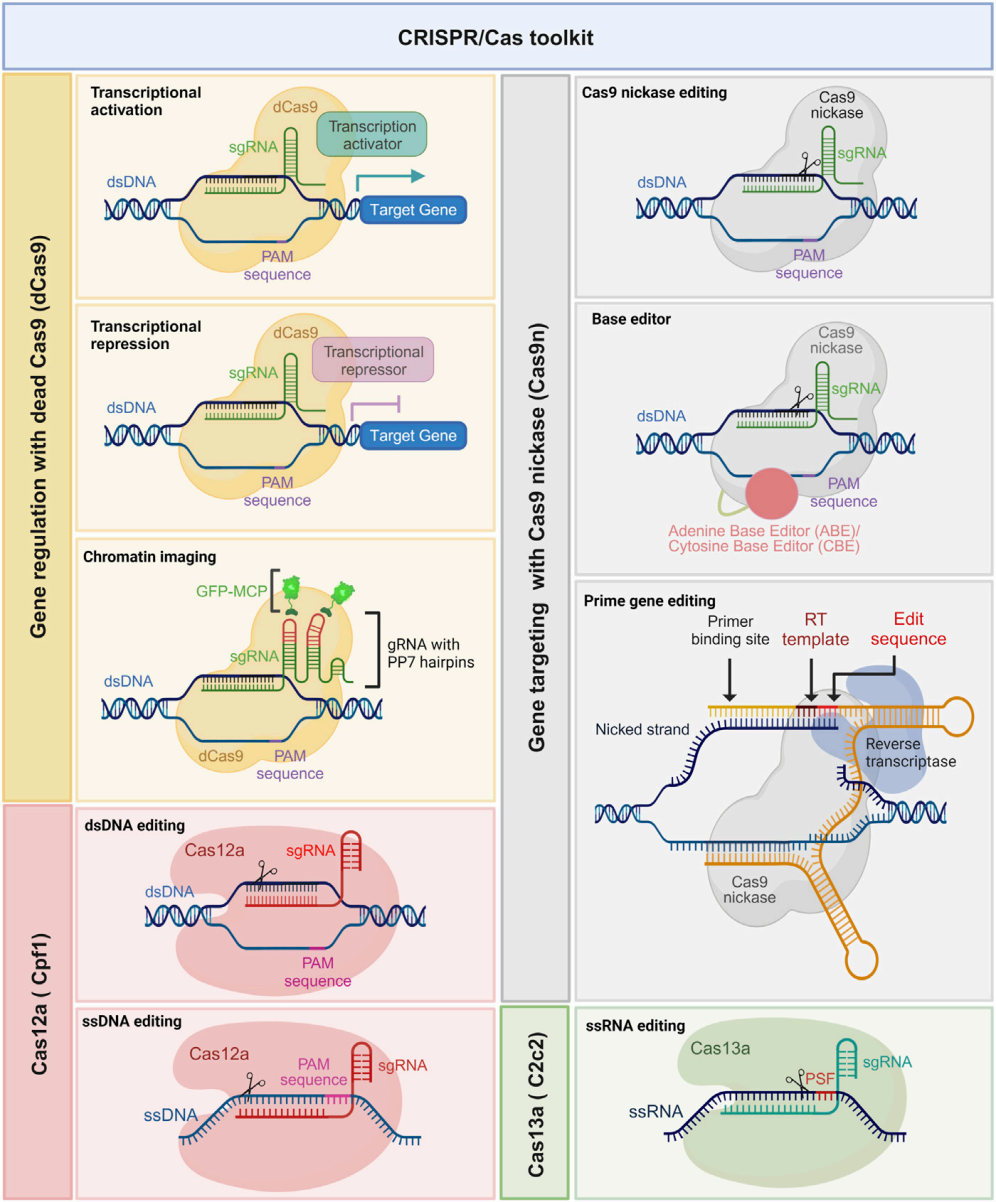
Expanding the potential of the CRISPR/Cas toolkit. Leveraging catalytically dead Cas9 (dCas9) (yellow boxes) and nickase Cas9 (nCas9) (gray boxes) with tailored modulators. This powerful combination allows for a multitude of functionalities, such as precise base editing using adenine base editor (ABE) and cytidine base editor (CBE), prime gene editing, intricate chromatin imaging, and fine-tuned transcription regulation. Additionally, Cas12a (pink boxes) facilitates editing of single-stranded DNA (ssDNA), while Cas13a (green boxes) enables the manipulation of single-stranded RNA (ssRNA). These innovative techniques significantly expand the toolkit’s applications, promising groundbreaking advancements in genetic research and manipulation.

The CRISPR gene editing revolution initially began with the use of wild-type Cas9. However, the field has rapidly evolved, and several modified versions of Cas enzymes have emerged with enhanced functionalities (Mougiakos et al., 2016). These modified Cas enzymes, also known as Cas variants or orthologues, possess distinct properties such as altered PAM specificities (Kleinstiver et al., 2015; Hu et al., 2018), enhanced DNA cleavage activity, expanded target range, and improved target specificity primarily through reduction of off-target effects (Figure 2) (Slaymaker et al., 2016). For instance, Cas9 variants such as Cas9 nickases (Cas9n), high-fidelity Cas9s (e.g., SpCas9-HF), and small Cas9s (e.g., SaCas9) have been engineered to reduce off-target effects and enhance precision (Trevino and Zhang, 2014; Kim et al., 2017). Moreover, novel Cas enzymes such as Cas12a (formerly known as Cpf1) and Cas13a (formerly known as C2c2) have been discovered and repurposed for RNA-guided DNA and RNA editing, respectively (Zetsche et al., 2015; Ishino et al., 2018).

The CRISPR/Cas9 system offers the ability to not just disrupt targeted genes but also modify them for epigenetic modulation (Xie et al., 2018). This versatility allows for the upregulation (termed CRISPR activation or CRISPRa) or downregulation of gene expression. To achieve these modifications, Cas9 is transformed into a deactivated form known as dead Cas9 (dCas9), where both endonuclease domains are artificially rendered non-functional, eliminating its ability to create double-stranded breaks. Subsequently, transcriptional regulators are either directly fused with dCas9 or linked through peptide sequences, enabling the recruitment of the cell’s transcriptional machinery. This recruitment leads to the assembly of RNA polymerase and other relevant factors at the target site, ultimately triggering the induction of gene transcription.

A similar strategy was employed to achieve downregulation of gene expression, where transcription suppressors are fused with dCas9, effectively obstructing the transcription pathway (Armando Casas-Mollano et al., 2020). In this context, the transcriptional activator VP64, consisting of four copies of the VP16 viral protein sequence (known for its transcriptional activation properties), and the suppressor KRAB (Krüppel-associated box), comprising approximately 75 amino acid residues (with a minimal module of about 45 residues), are both fused with dCas9. By providing a target site through the single-guide RNA, dCas9 orchestrates either transcriptional activation or suppression (Armando Casas-Mollano et al., 2020). This modification, known as epigenome editing, plays a crucial role in regulating altered gene expression in cancer cells.

Furthermore, another modification involves deactivating one of the two endonuclease domains of Cas9, resulting in a modified form called nickase Cas9 (nCas9). This nCas9 is particularly valuable for introducing paired nicks and enhancing cleavage specificity (Lee et al., 2023). Impaired Cas9 (nCas9 or dCas9), employed by David Liu’s group, has been utilized for various purposes. One such application involves generating cytidine base editors (CBE) and adenine base editors (ADE) for nucleotide substitutions from C-to-T and A-to-G, respectively (Komor et al., 2016; Gaudelli et al., 2017). Also, nCas9 fused with a reverse transcriptase enzyme, along with a prime editing guide RNA (pegRNA), enables precise insertion, deletion, or substitution of specific DNA sequences at the target site (Anzalone et al., 2019). Additionally, it has proven highly efficient in live-cell imaging CRISPR tools with single or multiple fluorescent proteins, allowing a deeper understanding of chromatin structure (Qin et al., 2017), chromatin dynamics (Dreissig et al., 2017), and topology through manipulation of chromatin loops between regulatory genomic regions (Morgan et al., 2017). In a novel study, G. Yu and others extensively characterized the factors affecting prime editing efficiency and developed computational models for tailored, large scale and error-free activity prediction in multiple cell types and for different prime editors (Yu et al., 2023). Length, GC content and melting temperature of the primer binding site (PBS) and the reverse transcription template (RTT), editing position, minimal length of the right homology arm (RHA), number and preferred type of edited nucleotides, among other determinants, were investigated in depth and proved to have a crucial impact on prime editing efficiency. Analog to the optimization of the sgRNA scaffold for enhanced Cas9 activity, researchers demonstrated the feasibility of improving prime editing efficiency using an optimized pegRNA scaffold. These models, termed DeepPrime, are a collection of 18 different tools based on convolutional neuronal networks (CNN) and were proven to be very well suited for the generation and correction of pathogenic mutations reported in large ClinVar datasets. DeepPrime was also shown to be useful for reducing off-target effects and prioritizing the target sequences to be tested. Such computational models remain to be tested for further cell types, for instance, primary cultured cells and pluripotent stem cells, and expanded to other different delivery methods.

Further cutting-edge computational models and algorithms have made major contributions to the advancement of CRISPR/Cas-libraries with vast potential for their application for *in vivo* therapy. The advantage of using genome-wide knockout libraries for the identification of essential genes for different cell types is well known. Yet, the computational analysis of the data generated by these screens turns out to be complex and challenging. For instance, Li *et al.* proposed a computational algorithm termed Model-based Analysis of Genome-wide CRISPR/Cas Knockout (MAGeCK) to identify and prioritize not only essential sgRNA and genes but also relevant pathways for specific cell types. By ranking sgRNAs based on *p*-values calculated from a negative binomial model, this statistical method showed significantly higher sensitivity and robustness compared with other algorithms (Li et al., 2014).

Similarly, further studies have been focused on the development of computational models to optimize the sgRNA design and maximize the activity of the CRISPR/Cas-system in functional knockout screens, while minimizing potential off-target effects. Some of these approaches rely on computing the log odds ratio of nucleotide frequency between target DNA sequences (Xu et al., 2015), on improving sgRNA design rules for on- and off-target activity (Doench et al., 2016) or on deep learning-based training (Kim et al., 2019) to develop powerful activity-predicting models with broad applicability based on large datasets.

### 2.3 Comparison of CRISPR/Cas with other gene editing methods such as TALENs and ZFNs

Unlike traditional gene editing methods, such as TALENs (Transcription Activator-Like Effector Nucleases) and ZFNs (Zinc Finger Nucleases), CRISPR offers the advantage of enabling the precise and permanent integration of a corrected or missing gene into the host genome through a single genome editing event (Gaj et al., 2013).

The advantages of CRISPR over TALENs and ZFNs lie in its high accuracy of target gene recognition owing to the Watson-Crick base pairing between guide RNA and target DNA, reduced risk of oncogene activation and minimized mutagenesis (Boettcher and McManus, 2015). Additionally, CRISPR eliminates the need for repeated dosing in certain applications, although repeated administration of the *CRISPR/Cas9* system has been shown to enhance genome editing efficiency in some preclinical studies. Furthermore, CRISPR technology presents an advantage by reducing the necessity for frequent administration in certain applications. It should be noted, however, that certain preclinical research has indicated that repeated utilization of the CRISPR/Cas9 system can enhance the efficiency of genome editing (Kenjo et al., 2021). The use of CRISPR *in vivo* genome editing also offers a more rapid and cost-effective approach compared to traditional methods due to its practicability, versatility, and accuracy. This enables researchers and clinicians to advance their work in the development of new therapies for a variety of human diseases.

## 3 General applications of CRISPR/Cas genome editing in human health

**FIGURE 3:**
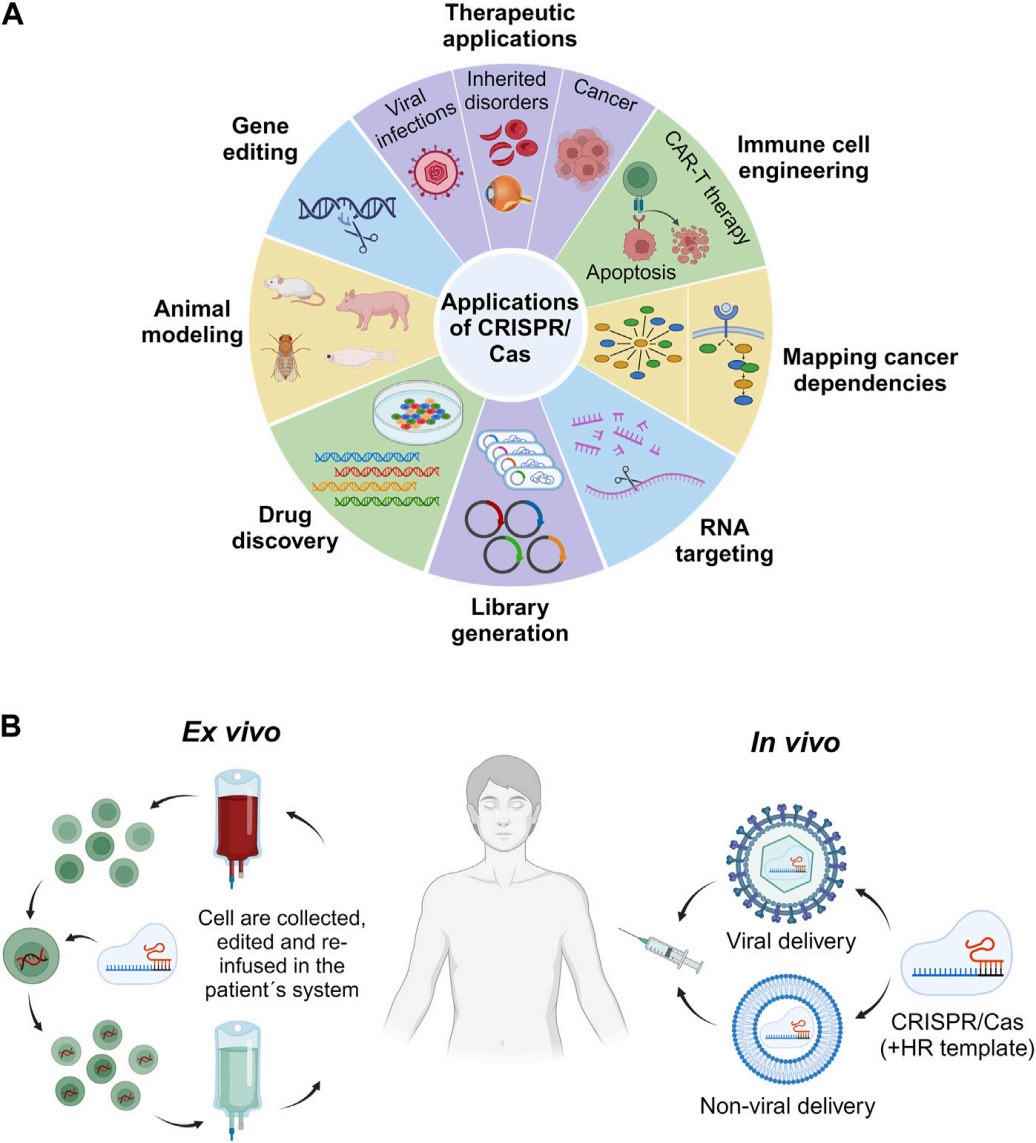
Applications of CRISPR/Cas. Overview of some of the most relevant biomedical and research fields for employment of the CRISPR/Cas technology **(A)**. Comparison of *in vivo* and *ex vivo* CRISPR/Cas-based therapeutic strategies. *In vivo* approaches involve the delivery of the CRISPR/Cas system by dint of either a viral or a non-viral vehicle for direct administration into the patient. The HR template denotes the homologous recombination template. By contrast, *ex vivo* strategies comprise the collection, *in vitro* editing via CRISPR/Cas, and re-infusion of cells into the patient **(B)**.

This section of the review focuses on the broad spectrum of CRISPR/Cas genome editing applications in the context of human health (Figure 3A). Specifically, we will explore the realm of *in vivo* applications, analyzing both the accomplishments and challenges associated with *ex vivo* CRISPR/Cas therapy. Furthermore, we will provide a comprehensive overview of the specific diseases and conditions that are currently under investigation for potential treatment through CRISPR/Cas therapy within the human body in general.

Due to its complexity and medical significance, we will discuss the applications of CRISPR/Cas genome editing of cancer in further detail in Section **Fehler! Verweisquelle konnte nicht gefunden werden.**

### 3.1 Success and limitations of *ex vivo* CRISPR/Cas therapy

CRISPR offers two distinct therapeutic approaches for treating genetic disorders. One avenue entails *ex vivo* genetic correction of patient cells, while the other involves *in vivo* modification of cells through targeted delivery of CRISPR effectors inside the patient’s body to specific tissues or organs (Figure 3B).

Despite the immense potential of *ex vivo* CRISPR/Cas9 trials and studies for improving human health, their limitations must be acknowledged. The feasibility of *ex vivo* gene therapy is dependent on the availability of cells, such as hematopoietic stem cells (HSCs), T cells, and natural killer (NK) cells, that can be conveniently harvested, genetically altered *in vitro*, propagated, and reintroduced into the patient’s body (Ying et al., 2019; Koniali et al., 2021; Elmas et al., 2022). Although *ex vivo* gene therapy has demonstrated clinical efficacy in the treatment of hematological diseases, the therapeutic utility is substantially hindered by the unsuitability of several tissues and organs difficult to access for this approach, thereby impeding its application in the management of various disorders.

On the other hand, the use of *in vivo* CRISPR/Cas9 technology has the potential to revolutionize the way we treat a range of diseases and conditions. Researchers are investigating the ability of CRISPR/Cas9 to precisely edit genes and address specific mutations and the potential to provide new cures and treatments for previously untreatable conditions (Yang T. C. et al., 2020; Abdelnour et al., 2021). Exciting clinical trials and studies are underway to evaluate the potential of *in vivo* CRISPR/Cas9 therapy. The ability of CRISPR/Cas9 to precisely edit genes and address specific mutations has the potential to provide new cures and treatments for sickle cell anaemia, beta-thalassemia (Shariati et al., 2016; Xu et al., 2021), and specific forms of inherited retinal disease (Abdelnour et al., 2021). Furthermore, researchers are exploring the ability of CRISPR/Cas9 to target cancer cells and enhance the effectiveness of cancer immunotherapy. The use of CRISPR/Cas9 to treat viral infections, such as human immunodeficiency virus (HIV) (Bhowmik and Chaubey, 2022) and hepatocellular carcinoma associated with hepatitis B virus (HBV) infection (Yang Y. C. et al., 2020), is also attracting attention.

*Ex vivo* therapeutic gene editing mediated by the CRISPR/Cas system has witnessed unprecedented success in chimeric antigen receptor (CAR)-T therapy (Ying et al., 2019; Sterner and Sterner, 2021). Nevertheless, when scaled up to the clinical level, this procedure is laborious, time-consuming, expensive, and has limited scalability. The direct administration of CRISPR/Cas9 to targeted cells for gene editing could pave the way for the *in vivo* application of CRISPR as a therapeutic tool in patients.

### 3.2 Overview of *in vivo* applications

A groundbreaking step forward in gene therapy has been initiated in recent years through the inception of a clinical trial utilizing *in vivo* delivery of CRISPR/Cas9, for the first time in patients, marking an exciting development in the field (Table 1). Due to its accessibility and the tight blood-ocular barriers, the eye was among the first organs used for gene therapy studies. One such retinal disorder is Leber congenital amaurosis (LCA), a monogenic disorder that leads to childhood blindness due to a bi-allelic loss-of-function mutation in the CEP290 gene. LCA has no current treatment options and can significantly impair one’s quality of life. One such deleterious intronic IVS26 mutation involving c.2991 + 1655A>G in intron 26 of the CEP290 gene. This mutation triggers aberrant splicing leading to a non-functional protein. EDIT-101, a therapeutic approach, delivers CRISPR/Cas9 directly to the retina of individuals with LCA targeting the intronic IVS26 mutation (Maeder et al., 2019). The adeno-associated virus serotype 5 (AAV5) was employed as a viral delivery system to transport the *staphylococcus aureus* Cas9 enzyme and meticulously designed guides, known for their high activity and specificity. The objective was to target the IVS26 mutation within the CEP290 gene. By utilizing AAV5, the genetic components could be efficiently introduced into the target cells, leveraging the viral system’s ability to penetrate host cells and deliver the desired genetic material precisely to the intended genomic site. A subsequent double-stranded break is induced, leading to a deletion or inversion of the IVS26 region, preventing aberrant splicing and generation of a fully functional protein.

**TABLE 1:** Interventional clinical trials registered for CRISPR/Cas9-based gene editing.

NCT no.	Target gene and effect	Disease	Intervention	Phase	Country
NCT03398967	Cas9-mediated creation of CD19 and CD20 or CD19 and CD22 CAR-T cells	B cell leukemia, B cell lymphoma	Universal Dual Specificity CD19 and CD20 or CD22 CAR-T Cells	Phase 1/2	China
NCT03164135	CCR5 knockout	HIV-1-infection	Modified CD34^+^ hematopoietic stem cells	Not applicable	China
NCT03728322	Correction of the hemoglobulin subunit β globulin gene	Thalassemia	*Ex vivo* modified hematopoietic stem cells for treatment	Early phase 1	United States
NCT04035434	Creation of a CD19-directed CAR-T cell	B-cell malignancy	CTX110, CD19-directed T-cell immunotherapy	Phase 1	United States, Australia, Canada, Germany, Netherlands, Spain
NCT04244656	CTX120 B cell maturation antigen (BCMA)-directed T cell immunotherapy	Multiple myeloma	Biological safety and efficacy of CTX120 in multiple myeloma	Phase 1	United States
NCT04438083	CTX130 CD70-directed T cell immunotherapy comprised of allogeneic T cells	Renal cell carcinoma	Safety and efficacy of CTX130 in relapsed or refractory renal cell carcinoma	Phase 1	United States, Netherlands
NCT04426669	Cytokine-induced SH2 (CISH) protein inhibition	Gastrointestinal epithelial cancer	Tumor-infiltrating lymphocytes (TILs) inhibited immune checkpoint CISH, combined with Cyclophosphamide, Fludarabine, Aldesleukin	Phase 1/2	Minnesota
NCT03655678	Disruption of the erythroid enhancer to BCL11A gene	Beta-Thalassemia	CTX001, *ex vivo* modified hematopoietic stem cells	Phase 2/3	United States
NCT03745287	Disruption of the erythroid enhancer to BCL11A gene	Sickle cell disease	CTX001, *ex vivo* modified hematopoietic stem cells for treatment	Phase 2/3	United States, Germany, Italy, France, Belgium
NCT03057912	E6 and E7 oncogene of HPV16 and HPV18 deletion	Human Papillomavirus-related malignant neoplasm	Safety and efficacy study of TALEN and CRISPR/Cas9 in the treatment of HPV-related cervical intraepithelial neoplasia	Phase 1	China
NCT04417764	PD-1 knockout engineered T cells	Advanced hepatocellular carcinoma	Transcatheter arterial chemoembolization combined treatment with PD-1 knockout engineered T cells to block the blood supply of the tumor	Phase 1	China
NCT03545815	Programmed cell death protein 1 (PD-1) and TCR knockout	Solid tumor, adult	Anti-mesothelin CAR-T cells with added PD-1 and TCR knockout	Phase 1	China
NCT03747965	Programmed cell death protein 1 (PD-1) knockout	Solid tumor, adult	Mesothelin-directed CAR-T cells	Phase 1	China
NCT03081715	Programmed cell death protein 1 (PD-1) knockout	Esophageal cancer	Modified PD-1 knockout T Cells	Not applicable	China
NCT02793856	Programmed cell death protein 1 (PD-1) knockout	Metastatic non-small cell lung cancer	Cyclophosphamide combined with PD-1 Knockout T Cells	Phase 1	China
NCT04037566	Refractory B cell malignancies	Acute lymphocytic leukemia (ALL) in relapse	XYF19, CD19-CAR modified T cells with CAR delivered by lentivirus and Cas9 knockout of HPK1 combined with Cyclophosphamide and Fludarabine	Phase 1	China
NCT03166878	βTCRα, TCRβ, β-2 microglobin (B2M) knockout	B cell leukemia, B cell lymphoma	Safety and tolerability of Universal gene-disrupted allogeneic CD19-directed BBζ CAR-T cells CAR-T cell (UCART019) in patients	Phase 1/2	China
NCT04767308	βTCRα, TCRβ, β-2 microglobin (B2M) knockout	CD5^+^ relapsed/refractory hematopoietic malignancies	CD19-CAR-modified T cells with CAR delivered by lentivirus and Cas9 knockout B2M and TCR to create universal T cells	Early phase 1	NA
NCT04560790	HSV-1 genome clearance	Viral Keratitis	BD111, CRISPR/Cas9 mRNA single dose was injected in the cornea of adult	Not applicable	China
NCT05210530	Generate pancreatic beta cells that can evade the immune system	Type 1 Diabetes Mellitus	VCTX210A comprises 2 components 1) allogeneic pancreatic endoderm cells (PEC210A) genetically modified with CRISPR to promote immune evasiveness and survival 2) perforated device designed to deliver and retain the PEC210A cells	Phase 1	Canada
NCT05565248	Generate pancreatic beta cells that can evade the immune system	Diabetes Mellitus	VCTX211, CRISPR/Cas9 modified PEC211 cells loaded into a delivery device and implanted in subjects	Phase 1/2	Canada
NCT04990557	PD-1 and ACE2 Knockout	COVID-19 Respiratory Infection	PD-1 and ACE2 Knockout T Cells will be expanded *ex vivo* and infused back to the patients for treatment	Phase 1/2	NA
NCT05566223	Inactivation of gene Encoding CISH	Carcinoma, non-small-cell lung cancer	CISH-inactivated tumor-infiltrating lymphocytes (TIL) administration to subjects in combination with checkpoint inhibitors	Phase 1/2	United States
NCT05477563	Disruption of the erythroid enhancer to BCL11A gene	Beta-thalassemia or severe sickle cell disease	Efficacy and safety of a single dose of autologous CRISPR Cas9 modified CD34^+^ human hematopoietic stem and progenitor cells (hHSPCs) (CTX001) in subjects	Phase 3	United States, Italy
NCT05795595	CD70-directed T cell immunotherapy comprised of allogeneic T cells that incorporate novel Regnase-1 and TGFBR2 edits	Clear cell renal cell carcinoma, cervical carcinoma, esophageal carcinoma, pancreatic adenocarcinoma, malignant pleural mesothelioma	Safety and efficacy of CTX131 (CD70-directed T cell immunotherapy comprised of allogeneic T cells) in subjects with relapsed or refractory solid tumors	Phase 1/2	United States
NCT05643742	CD19-directed T cell immunotherapy comprised of allogeneic T cells that incorporate novel Regnase-1 and TGFBR2 edits	B-cell lymphoma	Safety and efficacy of CTX112 in subjects with relapsed or refractory B-cell malignancies	Phase 1/2	United States
NCT04502446	CD70-directed T-cell immunotherapy comprised of allogeneic T cells genetically modified *ex vivo* using CRISPR-Cas9	T or B Cell lymphoma	Safety and efficacy of CTX130 in subjects with relapsed or refractory T or B cell malignancies	Phase 1	United States, Australia, Canada
NCT04637763	CRISPR-Edited (PD-1 knock out) Allogeneic Anti-CD19 CAR-T Cell Therapy	Lymphoma, Non-Hodgkin	Safety, efficacy, and immunogenicity of CB-010 in adults with relapsed/refractory B cell non-Hodgkin lymphoma after lymphodepletion consisting of cyclophosphamide and fludarabine	Phase 1	United States
NCT05722418	CRISPR-edited allogeneic anti-BCMA CAR-T cell	Relapsed/refractory multiple myeloma	Safety of CB-011 in subjects with relapsed or refractory multiple myeloma	Phase 1	United States
NCT04925206	CRISPR-Cas9-modified CD34^+^ human hematopoietic stem and progenitor cells (hHSPCs)	Transfusion dependent Beta-thalassemia	Safety and efficacy of ET-01 in subjects with transfusion-dependent Beta-thalassemia	Phase 1	China
NCT04557436	Anti-CD19 universal CAR-T cell therapy with knockout of CD52 and TRAC	B-cell acute lymphoblastic leukaemia (B-ALL)	PBLTT52CAR19, CAR19 universal T cells for treatment of children with relapsed or refractory B-ALL	Phase 1	United Kingdom
NCT05356195	Disruption of the erythroid enhancer to BCL11A gene	Beta-thalassemia	Evaluate efficacy and safety of a single dose of Autologous CRISPR Cas9 Modified CD34^+^ hHSPCs (CTX001) in Pediatric Subjects with transfusion-dependent β-thalassemia	Phase 3	United States, Italy, United Kingdom
NCT05329649	Disruption of the erythroid enhancer to BCL11A gene	Sickle cell disease, dydroxyurea intolerance	Safety and efficacy of autologous CRISPR-Cas9 modified CD34^+^ hHSPCs (CTX001) in pediatric participants with severe SCD and hydroxyurea (HU) failure or intolerance	Phase 3	United States
NCT05577312	Disruption of the erythroid enhancer to BCL11A gene	Beta-thalassemia	Safety and efficacy of intravenous infusion of autologous CRISPR-Cas9 modified CD34^+^ hHSPCs (BRL-101) in Subjects with transfusion-dependent β-thalassemia (TDT)	Phase 1	China
NCT05144386	Gene disruption of three undisclosed genomic sites in the HIV DNA	HIV-1-infection	Safety, tolerability, and biodistribution of EBT-101 in aviremic HIV-1-infected adults on stable antiretroviral therapy	Phase 1	United States
NCT05662904	CD33 deletion	Relapsed/refractory acute myeloid leukemia (AML)	Donor-derived CD34^+^ HSC with CRISPR/Cas9-mediated CD33 deletion combined with Gemtuzumab and Ozogamicin	Phase 1	Germany
NCT04601051	TTR knockout	Transthyretin-related (ATTR) familial amyloid polyneuropathy	Safety, tolerability, pharmacokinetics (PK), and pharmacodynamics (PD) of NTLA-2001 in participants with hereditary transthyretin amyloidosis with ATTR polyneuropathy or cardiomyopathy	Phase 1	France, New Zealand, Sweden, United Kingdom
NCT05631912	T-cell receptor α constant (TRAC) disruption and anti-CD19 Synthetic T-cell Receptor Antigen Receptor T (STAR-T) knock in	Non-Hodgkin lymphoma (NHL), B Cell	Autologous TRAC locus-inserted CD19-targeting STAR-T cells for relapsed and refractory B-cell NHL combined with Fludarabine and Cyclophosphamide	Phase 1/2	China
NCT04976218	Transforming growth factor-β (TGF-β) receptor II knockout	Solid tumor, adult	Safety profiles of CAR-EGFR-TGFβR-KO T cell in the treatment of previously treated advanced EGFR antigen overexpressing solid tumors	Phase 1	China
NCT05397184	Cryopreserved base edited (BE) CAR7 T cells (BE752TBCCLCAR7PBL)	Relapsed/refractory T-cell acute lymphoid leukaemia	BE CAR7 T cells (BE752TBCCLCAR7PBL) to treat T cell malignancies in children	Phase 1	United Kingdom
NCT05143307	Gene disruption of three undisclosed genomic sites in the HIV DNA	HIV-1-infection	Long-term follow-up study of HIV-1-infected adults who received EBT-101	Phase 1	United States
NCT05120830	Inactivate kallikrein B1 (KLKB1)	Hereditary angioedema (HAE)	Safety, tolerability, pharmacokinetics, and pharmacodynamics of NTLA-2002 in adults with HAE via normal saline IV administration	Phase 1/2	France, United Kingdom, Netherlands, New Zealand, United Kingdom
NCT05812326	PD-1 knockout	Advanced breast cancer	AJMUC1-PD-1 gene knockout anti-MUC1 CAR-T cells in the treatment of advanced MUC1-positive breast cancer	Phase 1/2	China
NCT03044743	PD-1 knockout	Stage IV gastric carcinoma; Stage IV nasopharyngeal carcinoma; T cell lymphoma stage IV; T cell lymphoma stage IV; stage IV DLBCL	PD-1 Knockout EBV-CTLs for Advanced Stage Epstein-Barr Virus (EBV) Associated Malignancies combined with Fludarabine; Cyclophosphamide; Interleukin-2	Phase 1/2	China
NCT03872479	gRNAs targeting the IVS26 mutation in the CEP290 gene	Leber congenital Amaurosis 10; inherited retinal dystrophies	Single ascending dose study in participants with LCA10	Phase 1/2	United States
NCT05444894	Edit the promoter regions of gamma-globin gene 1 and 2 to increase the expression of HbF	Transfusion dependent Beta thalassemia; hemoglobinopathies; Thalassemia major; Thalassemia intermedia	EDIT-301 for autologous hematopoietic stem cell transplant (HSCT) in participants with transfusion-dependent beta thalassemia (TDT)	Phase 1/2	United States
NCT05514249	Upregulate an alternate isoform of the dystrophin protein	Duchenne muscular dystrophy	Treatment of a single patient with a single dose of CRD- TMH-001 via intravenous injection	Phase 1	

The patients were given a one-time dose of EDIT-101 through a subretinal injection, which is targeted directly to photoreceptor cells in one of their eyes. The treatment was well-tolerated without any severe adverse events or dose-limiting toxicities in the eyes. The adverse events observed were mostly mild and in line with what was expected for subretinal delivery. The 17 November 2022 report by Editas Medicine shows that out of the 14 participants who underwent the treatment, three individuals demonstrated a significant improvement in their best corrected visual acuity (BCVA). Moreover, these participants showed consistent improvement in two of the following measures: full field sensitivity test (FST), visual function navigation course (VFN), or visual function quality of life (VFQ). Phase 1/2 BRILLIANCE trial of EDIT-101 (NCT03872479) is ongoing in up to 34 LCA patients who will be monitored for 3 years. The promising outcomes of these clinical trials serve as a confirmation of the concept behind the gene editing platform, specifically its ability to be effective *in vivo*. The positive results obtained from these clinical trials represent a significant milestone in demonstrating the feasibility of *in vivo* CRISPR/Cas base gene editing platforms.

Another sublime example is provided by Gillmore *et al.*, who conducted a preclinical investigation (Intellia Therapeutics, 2020) which showcases the significant efficacy and safety of NTLA-2001, a novel *in vivo* CRISPR/Cas9-mediated gene therapy for the treatment of Transthyretin (TTR) Amyloidosis, also called ATTR Amyloidosis (Gillmore et al., 2021). Hereditary ATTR (hATTR) amyloidosis, a less common form of ATTR amyloidosis, can be caused by more than 100 different pathogenic mutations in the TTR gene (Ando et al., 2013). This disease is a fatal and advancing condition distinguished by an abnormal accumulation of amyloid fibrils composed of misfolded transthyretin (TTR) protein in the body’s tissues and organs. It is increasingly acknowledged as a contributor to polyneuropathy with or without cardiomyopathy including heart failure.

The primary objective of this research was to assess the effectiveness of a single ascending dosage of NTLA-2001 in treating patients with polyneuropathic hATTR amyloidosis through TTR editing and knockout in human hepatocytes (Gillmore et al., 2021). Lipid nanoparticles (LNPs) served as the delivery system using intravenous IV) administration. This preclinical trial demonstrated that a single dosage of 0.3 mg NTLA-2001 per kilogram resulted in knockout and an average reduction of 87% in serum TTR protein among the participants, meeting the clinical effectiveness standards. Crucially, the study participants showed good tolerance and safety to NTLA-2001. The only side effects that occurred were mild and included headaches, nausea, infusion-related reactions, decreased thyroxine levels, and rhinorrhea. This study had a comparatively small sample size of six patients, and the duration of the follow-up period was restricted to 28 days. To validate the sustainability of the therapeutic outcomes and to ensure safety, it is imperative to conduct a prolonged follow-up on a larger cohort of patients with TTR amyloidosis. Indeed, the second phase of the clinical trial is presently underway, which continues to determine the appropriate dosage for the cohort expansion, including cardiomyopathy patients. Overall, this study showcases the potential of gene therapy for inherited diseases using the LNP-CRISPR system and evokes a positive outlook.

Intellia Therapeutics has undertaken another pivotal investigation involving the development of NTLA-2002 (Seitzer, 2021) (ClinicalTrials.gov number NCT05120830), a therapeutic intervention aimed at treating hereditary angioedema (HAE), a genetic disorder characterized by recurrent episodes of localized subcutaneous or submucosal swelling. Current HAE prophylactic treatments require chronic, lifelong administration of preventive medications including monoclonal antibody therapies. NTLA-2002, a systemically administered, single-dose CRISPR/Cas9-mediated therapy, has been engineered to selectively target and knocknock out KLKB1 gene in the liver, which is responsible for encoding the precursor protein prekallikrein. NTLA-2002 is delivered as a Cas9 mRNA and gRNA via lipid nanoparticles. Through intravenous infusion, NTLA-2002 acts to modulate plasma kallikrein activity and thereby mitigate the frequency and severity of HAE attacks. Studies in monkeys have confirmed these reductions have been sustained for at least 15 months. The ongoing human clinical trial testing different dose levels of NTLA-2002 exhibited a favorable safety profile, with the majority of observed adverse events being mild in nature, with no clinically significant laboratory abnormalities. This provides promising evidence for its potential as a one-time treatment for patients with HAE. Intellia is conducting Investigational New Drug (IND)-enabling activities for NTLA-3001 and NTLA-2003 for lung and liver-related diseases, respectively.

## 4 CRISPR/Cas-mediated therapy applications in cancer

Cancer is one of the leading causes of death accounting for around 10 million deaths worldwide (Bray et al., 2021; Sung et al., 2021). Cancer initiation and progression are driven by a complex interplay of genetic and epigenetic changes that occur at various levels, including nucleotide, gene, chromatin, and cellular levels (Lu et al., 2020). These gene mutations result in the dysregulation of key cellular processes and the acquisition of hallmark cancerous traits, leading to fitness advantages that promote tumor formation. Somatic cells exhibit a significantly elevated mutation rate, surpassing that of germline cells by up to one or two orders of magnitude (Flannery et al., 2021). Analysis of cancer genomes through next-generation sequencing has revealed an abundance of mutations in genes and epigenetic modifications within human tumors.

**FIGURE 4:**
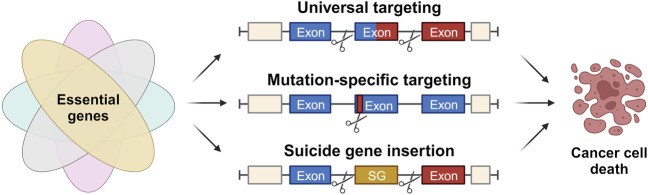
Approaches for application of CRISPR/Cas in cancer therapy. Strategies for application of the CRISPR/Cas system in cancer therapy based on the identification and targeting of genetic events solely present in malignant cells, eventually causing their specific cell death. These strategies include universal targeting of introns flanking the breakpoint region within a fusion oncogene, patient-specific targeting of mutations present in functional gene regions and the site-specific insertion of suicide genes (SG).

Despite the existence of various conventional cancer therapies, such as surgery, chemotherapy, and radiation therapy, their efficacy is often limited by their associated toxicities, which pose significant challenges to patients’ adherence and quality of life (Flannery et al., 2021). Molecular targeted therapy has emerged as a promising alternative to traditional chemotherapy, with high specificity and efficacy against certain tumor types. However, the high costs and limited long-term benefits of these drugs remain major drawbacks (Saijo, 2012). Immunotherapy, including immune checkpoint inhibitors, adoptive cell transfer, and tumor-specific vaccines, has also shown great promise in activating tumor-specific immune responses and suppressing cancer progression. Nonetheless, the efficacy of immunotherapy varies among different cancers and individuals, and immune escape mechanisms can limit its effectiveness. The precise and versatile genome-editing capabilities of the CRISPR/Cas9 technology hold great promise to overcome the obstacles and limitations currently associated with targeted cancer therapies to develop more effective and less toxic treatments for cancer. This section explores CRISPR/Cas9-based strategies for non-patient-specific universal targeting, patient-specific mutation targeting, and Cas9-mediated suicide gene insertion to combat cancer (Figure 4).

### 4.1 Non-patient-specific universal targeting

The process of oncogenesis involves an intricate cumulation of genetic mutations and dysregulated expression of a multitude of genes, encompassing oncogenes, tumor suppressor genes, chemoresistant genes, metabolism-related genes, and cancer stem cell-related genes (Lee and Muller, 2010; Emran et al., 2022; Orsolic et al., 2023). The CRISPR/Cas9 gene editing system offers a highly promising targeted approach for cancer treatment. Its ultimate objective is to restrain tumor growth and progression, for instance by specifically addressing mutations and restoring normal expression of dysregulated tumor suppressor genes or by suppressing the activity of oncogenes. By targeting essential genes, multiple studies (Hart et al., 2015; Reddy et al., 2017; Behan et al., 2019) have demonstrated the inhibitory impact of CRISPR/Cas on cell proliferation, migration, and infiltration.

Another innovative approach includes the identification and targeting of genetic events solely present in malignant cells. One interesting study aimed to develop a CRISPR/Cas9-based approach for targeting fusion oncogenes (Fos), which are chimeric genes resulting from in-frame fusions of the coding sequences of two genes involved in a chromosomal rearrangement (Martinez-Lage et al., 2020). Fusion oncogenes represent common genetic alterations found in many types of cancer and constitute powerful drivers of tumor development. Because their expression is exclusive to cancer cells and their elimination induces cell apoptosis in fusion oncogene-driven cancers, Fos emerges as attractive therapeutic targets. However, specifically targeting the resulting highly variable chimeric product genes is challenging.

In this study, the authors devised a simple, efficient, and non-patient-specific gene-editing strategy to specifically disrupt Fos in cancer cells. The strategy targets two introns flanking all breakpoint regions in patients, making it a universal approach for all cancer cells harboring a given FO irrespective of the FO isoform or the patient-specific breakpoint. Hence, the intersections do not affect the exonic sequences or the expression of wild-type alleles involved in the rearrangement as it induces two targeted intronic DSBs in both genes implicated in the FO. This importantly produces a cancer cell-specific genomic deletion that is dependent on the presence of the FO and has no effect on wild-type gene expression in non-cancerous cells (Martinez-Lage et al., 2020).

In a proof-of-concept, the researchers focused on disrupting the EWSR1-FLI1 (EF) fusion, which acts as a dominant transcription factor in Ewing sarcoma. By specifically targeting this driver fusion, they were able to inhibit tumor cell growth both *in vitro* and in patient-derived xenograft (PDX) models of Ewing sarcoma. Furthermore, when the gene deletion strategy was combined with chemotherapy, a significant regression in tumor size was observed, highlighting its potential as a complementary treatment approach. The effectiveness of this approach was also confirmed in the context of the BCR-ABL1 fusion in chronic myeloid leukemia (CML). These findings demonstrate the broad applicability and therapeutic potential of the authors’ gene-editing strategy for disrupting Fos in various cancer types, offering a promising avenue for future research and clinical intervention.

### 4.2 Targeting patient-specific mutations

In cancer therapy, it is critical to selectively target the genome of tumor cells to induce their death. In recent years, significant progress has been made in understanding the genetic landscape of human cancer and the mutations driving tumorigenesis. The identification of targetable mutations within various pathways has led to the development of precision medicine strategies. A number of studies have successfully demonstrated the feasibility of selectively targeting cells harboring specific mutations, utilizing their unique genomic characteristics to trigger programmed cell death (Chen et al., 2020; Kwon T. et al., 2022). This approach holds great promise for selectively eliminating cancer cells while minimizing the potential impact on healthy cells.

In 2020, Chen *et al.* demonstrated the efficacy of CRISPR-mediated genome editing in selectively targeting chronic myeloid leukemia (CML), a prevalent form of leukemia in adults characterized by the presence of the Philadelphia chromosome (Ph) and resulting BCR-ABL oncoprotein. Despite the proven success of tyrosine kinase inhibitors (TKIs), e.g., imatinib, in the treatment of CML, a significant percentage of patients remain unresponsive to therapy. To address this, the researchers employed lentiviral-based CRISPR/Cas9 editing to specifically target the BCR-ABL junction *in vitro* and *in vivo* (Chen et al., 2020). Unlike tyrosine kinase inhibitors (TKIs), which solely ensure temporary inactivation of the BCR-ABL1 oncoprotein during treatment, CRISPR/Cas9 offers a unique advantage by providing the capability for permanent oncogene knockout through sequence-specific DNA cleavage, thus enabling the effective and long-lasting silencing of oncogenes in human cells. The precision of CRISPR/Cas9 in sequence-specific DNA cleavage enables the effective silencing of oncogenes in a manner that was previously impossible in human cells. The results show a notable decrease in the population of leukemia cells was observed in the ABL-targeted mice. These results underscore the potential of this approach as a viable alternative strategy for patients with imatinib-insensitive or -resistant CML.

Recently, an innovative approach by Kwon *et al.* (Kwon T. et al., 2022) called cancer-specific insertions–deletions (InDels) attacker (CINDELA) has been shown to induce targeted cancer cell death by simultaneous and multiple DNA double-strand breaks. In this method, _∼_10–50 sgRNAs were used to target cancer-specific mutations thus causing cell death. The CINDELA method was successfully applied to kill cancer cell lines, xenografted cancer cells in mice, patient-derived glioblastoma, and in a patient-derived xenograft (PDX) lung cancer model without impairing normal cells or affecting healthy mice. Increased cell death with a rise in CINDELA sgRNA numbers is likely due to amplified DNA double-strand breaks (DSBs) and extensive DNA damage.

This is feasible due to an intact cell death pathway. However, a large number of cancers show aberrant cell death mechanisms as tumor suppressors like p53 are heavily mutated. It may cause different DNA repair capacities and differential cell death effects, probably requiring several days for cell death to occur. Further investigation is necessary to determine the underlying molecular pathways involved in CINDELA-induced cell death and effectively target cancer cells with different genetic backgrounds. Moreover, there is a high risk of increased off-target effects due to the usage of multiple sgRNAs. These off-target effects on healthy cells need to be explored further.

### 4.3 Patient-specific targeting for Cas9-mediated suicide gene insertion

A novel and potentially effective strategy for targeted cancer cell eradication involves utilizing the CRISPR/Cas9 system to precisely insert a suicide gene at specific genomic loci (Chen et al., 2017). In this approach, guide RNAs were meticulously designed to direct Cas9 to cleave at the breakpoint sequences unique to the cancer cells. Subsequently, a repair template comprising a suicide gene flanked by sequences homologous to those surrounding the breakpoints was delivered, thereby triggering the insertion of the gene. The suicide gene employed in the study was the herpes simplex virus thymidine kinase (HSV-tk) gene, which renders cells susceptible to elimination by the commonly used antiviral drug, ganciclovir. Rigorous *in vitro* and *in vivo* experimentation was conducted using diverse cancer cell lines and mouse models of cancer, demonstrating the efficacy of this approach in selectively eradicating cancer cells while sparing normal cells. The authors propose that this innovative approach holds promise as a therapeutic strategy for cancer treatment, particularly in cases where cancer is driven by specific genomic rearrangements. However, further research is warranted to optimize the methodology and ensure its safety prior to clinical trials in humans.

In order to establish the robustness and applicability of the proposed approaches, future research directions should focus on validation in additional cancer cell lines and animal models to thoroughly assess their effectiveness and specificity. Furthermore, optimization of the CRISPR/Cas9 system should be pursued to enhance the efficiency and accuracy of the gene insertion process. It is imperative to conduct further characterization of potential off-target effects of the gene insertion approach to ensure that unintended mutations or other deleterious effects are minimized. Additionally, the development of strategies for *in vivo* delivery of the CRISPR/Cas9 system and DNA repair template to cancer cells necessitates further optimization and rigorous testing. These critical investigations will be essential in advancing the potential clinical translation of this promising approach for cancer therapy while safeguarding against any potential risks and limitations associated with the technique. Some of these aspects are further elaborated upon in subsequent sections of this review.

## 5 Opportunities and challenges of *in vivo* CRISPR/Cas applications in cancer

Tumor heterogeneity in resistance and recurrence represents a significant challenge in cancer treatment. The presence of diverse molecular and cellular characteristics within and between tumors creates a complex and dynamic environment that can impact the effectiveness of treatment strategies. Current therapies are sometimes the cause of resistance and cannot address resistance effectively due to the unique characteristics of the tumor microenvironment (TME), the development of drug-resistant cancer cells, and the presence of genetic mutations and alterations that contribute to tumor heterogeneity. While previous studies have identified various mutations and genetic alterations that contribute to tumor heterogeneity, understanding the specific mechanisms that drive resistance and recurrence requires further investigation. It is crucial to consider the various factors that contribute to heterogeneity, including genetic instability, epigenetic modifications, and microenvironmental factors. Additionally, the development of targeted therapies that can address the unique molecular and cellular characteristics of individual tumors is critical to improving patient outcomes. Advances in technologies such as single-cell sequencing and liquid biopsies are promising approaches to elucidating the heterogeneity and underlying mechanisms of resistance in cancer. Despite these challenges, continued research and development of novel therapeutic strategies hold promise for improving the prognosis of cancer patients with resistant and recurrent tumors.

### 5.1 Combination of CRISPR/Cas with other cancer therapies to enhance efficacy

Combination therapies that involve the use of CRISPR/Cas9 gene editing technology have emerged as a promising strategy to overcome the challenges associated with relapse and refractory cancers. CRISPR/Cas9 offers the ability to target specific cancer-causing genes precisely and efficiently, providing a potential solution to the issue of tumor heterogeneity and evolution. The combination of CRISPR/Cas9 with other treatment modalities, such as chemotherapy, targeted therapy, or immunotherapy, can further enhance the potential benefits of this technology. For instance, CRISPR/Cas9 can be used to disrupt genes that confer resistance to chemotherapy or targeted therapy, thereby sensitizing tumors to these treatments.

Loss-of-function CRISPR-based genetic screenings have revealed several genes involved in diverse biological processes that enhance the sensitivity of tumors to immunotherapy. In a study published in the journal Nature, researchers utilized CRISPR/Cas9 gene editing technology to identify potential targets for cancer immunotherapy (Manguso et al., 2017). The researchers injected a library of cancer cells that had been modified using CRISPR/Cas9 into mice and subsequently treated them with immune checkpoint inhibitors. The study revealed that deletion of genes involved in multiple diverse pathways, including NF-κB signaling, antigen presentation, and the unfolded protein response, sensitized melanoma tumors to immunotherapy. Among the targets identified, deletion of the protein tyrosine phosphatase PTPN2 in tumor cells showed particular promise by enhancing the efficacy of immunotherapy through its effect on interferon-γ-mediated antigen presentation and growth suppression. This study highlights the potential of CRISPR/Cas9 technology for identifying novel targets for cancer immunotherapy and sheds light on the mechanisms that underlie tumor sensitivity to immune checkpoint inhibitors. Similar approaches can be used for *in vivo* therapy.

Targeting patient-specific mutations in key regulatory genes such as PTPN2 or NF-κB using CRISPR/Cas9 gene editing technology can lead to the knockout of specific proteins in tumor cells. By subsequently treating these edited cells with immune checkpoint inhibitors, it is possible to further sensitize them to immunotherapy. These innovative combination therapies have the potential to revolutionize cancer treatment by providing a more precise and personalized approach, tailored to each patient’s unique genetic makeup. By leveraging the power of CRISPR/Cas9 and immune checkpoint inhibitors, clinicians can potentially enhance the efficacy of existing cancer treatments, while minimizing toxic side effects. The ultimate goal of this approach is to improve patient outcomes and quality of life while minimizing the risk of cancer relapse and recurrence.

Moving from the exploration of combination therapies involving CRISPR/Cas9 gene editing in enhancing cancer treatment efficacy, we now shift our focus to the multifaceted aspect of genetic heterogeneity and the potential of multiplexed CRISPR/Cas systems for targeted gene regulation. Understanding the intricate gene regulatory networks and their implications in cancer progression, including therapeutic resistance, phenotypic plasticity, and metastasis, highlights the need for advanced approaches that can effectively address the diverse alterations within tumors. Furthering multiplexed CRISPR/Cas systems can expand the gene modulation toolbox, providing versatile options for precise targeting and modulation of complex regulatory networks in various cancers.

### 5.2 Multiplexing CRISPR/Cas for multi-targeted regulation

Native CRISPR/Cas systems present in archaea and bacteria and inherently multiplexed, i.e., these organisms naturally encode several CRISPR arrays and even various Cas proteins that provide a memory after first encounter with invading agents. Therefrom, multiplexing CRISPR technologies imply the simultaneous expression of numerous gRNAs or Cas enzymes Click or tap here to enter text. Moving from single-guided approaches towards multiplexed strategies enables transcriptional regulation and multi-locus editing, vastly enhancing the panel of possible technological and medicinal applications.

One strategy for modifying the activity of Cas endonucleases is the mutation of specific amino acids. By introducing defined mutations, the cleavage activity of Cas9 and Cas12a, the most commonly utilized enzymes for transcriptional control and gene editing, can be abolished. The resulting nuclease-null mutants, known as dCas9 and dCas12a, can be fused to different effector domains for efficient transcriptional regulation, including CRISPR-mediated activation (CRISPRa) and inhibition (CRISPRi) (Bikard et al., 2013; Qi et al., 2021). Similar to the inactivated Cas9 approach, a Cas12a variant with Dnase-dead activity (ddCas12a) used along with crRNAs harboring mutated direct repeats has been demonstrated to allow quantitative regulation of multiple genes (Wang et al., 2019).

By means of these technologies, numerous gRNAs and Cas nucleases can be applied for simultaneous editing, activation, and inhibition of different target genes, eventually enabling a variety of applications such as control of cell fate and cellular behavior, modulation of metabolic pathways or combinatorial mapping of genotype-to-phenotype, among many others. Yet, in addition to the simultaneous targeting of multiple genomic targets, gene editing and transcriptional regulation can be significantly increased by directing several gRNAs to a single genetic locus (Cheng et al., 2013).

With rising numbers of gRNAs, additional control mechanisms to avoid unintended interactions between components and minimize off-target effects must be implemented. Employing various Cas orthologs, each recognizing a slightly different gRNA structure, using truncated gRNAs able to mediate transcriptional regulation, or establishing inducible systems for the regulated expression of the CRISPR/Cas elements, represent some control mechanisms to limit off-target effects when multiplexing CRISPR technologies (Bikard et al., 2013; Polstein and Gersbach, 2015; McCarty et al., 2020).

On the other hand, the heterogeneity within tumors of various cancers is marked by the activation of unique gene regulatory networks, shaped by a complex interplay of factors from the tumor microenvironment and tumor cell-intrinsic parameters, encompassing genetic mutations, epigenetic modifications, and transcriptional regulations (Marusyk et al., 2020). Blood cancers, including leukemia and lymphoma, exhibit distinct gene regulatory networks shaped by various factors mentioned above. Examples of specific regulatory networks in these diseases include the BCR-ABL1 network driving chronic myeloid leukemia (CML), dysregulated NOTCH signaling pathway in T-cell acute lymphoblastic leukemia (T-ALL), MYC regulatory network impacting multiple hematological malignancies, alterations in epigenetic modifiers, and dysregulated transcription factor networks. Building upon the understanding of diverse regulatory networks, it is crucial to recognize that the implications of genetic heterogeneity extend to various aspects of cancer progression.

The implications of genetic heterogeneity in cancer are multifaceted, encompassing a range of consequences. Diverse forms of heterogeneity, including epigenetic heterogeneity, may play pivotal roles in the acquisition of clinically significant traits, such as therapeutic resistance, metastatic dissemination, and phenotypic plasticity. Transcriptional regulation is a key to such processes. Notably, epigenetic heterogeneity, specifically in the form of DNA methylation, has been consistently observed in regulatory regions that modulate the transcription of genes associated with the disease process.

In this context, multiplexing CRISPR emerges as a promising strategy to overcome the challenges derived from tumor heterogeneity. For instance, the disruption of gene combinations relevant to a specific cancer type can rapidly shed light on their contribution to a particular phenotype. One unsophisticated and robust approach for dissecting the genotype-phenotype connection is the use of genetic barcodes linked to gRNA sequences, followed by repeated deep sequencing over time and analysis of the barcode frequencies over that period. Based on this concept, one study applied the so-called combinatorial genetics en masse (CombiGEM) technology to create a library of 23,409 barcoded dual gRNA pairs and determine gene combinations that prevent ovarian cancer cell growth upon disruption (Wong et al., 2016).

Together, these multiplexing CRISPR/Cas technologies provide powerful means for combinatorial and precise regulation of multiple genes or regulatory elements in cancer cells, opening up new avenues for developing targeted cancer therapies.

### 5.3 Co-targeting oncogenes and tumor suppressors: multiplexing CRISPR/Cas for overcoming PAM limitations

The main challenge in genome editing approaches is the limited availability of protospacer adjacent motifs (PAMs) in desired gene loci, which can restrict target selection. However, advancements in Cas-nuclease variants, such as Cas12a and Cas13, have expanded the options for genome editing by reducing PAM limitations. These Cas-nuclease variants offer increased flexibility for targeting specific sites in the genome.

One notable application of inactivated Cas13 is site-directed RNA engineering. In contrast to Cas12a, the endonuclease Cas13 does not require a PAM sequence to accomplish its RNAse activity, rendering the targeting capability of this protein almost unlimited (Abudayyeh et al., 2017). By fusing inactivated Cas13 with different enzymes, multiple strategies have been developed for precise RNA modification. Platforms such as “RNA editing for programmable A to I replacement” (REPAIR) (Cox et al., 2017), “RNA editing for specific C to U exchange” (RESCUE) (Abudayyeh et al., 2019), “C to U RNA editor” (CURE) (Huang et al., 2020), and “CRISPR/Cas-inspired RNA targeting systems” (CIRTS) (Rauch et al., 2019) have shown the successful fusion of Cas13 to various enzymes, usually specific deaminases, to induce target-oriented RNA exchanges, to deliver or remove RNA modifications or even to deliver multiple effector proteins, including translational activators or RNA degrading enzymes. These approaches have been employed to correct mutations in a number of proteins linked to disorders such as nephrogenic diabetes insipidus, Fanconi anaemia, retinal degeneration, Parkinson´s disease, or Duchenne muscular dystrophy, which have been thoroughly reviewed elsewhere (Khosravi and Jantsch, 2021).

Along with translocations, InDels, and missense mutations, a large number of tumor-associated alterations found in cancer patients are indeed single-nucleotide nonsense substitutions that create premature termination codons (PTCs) in tumor suppressor genes. Mutations in tumor suppressor genes can result in loss of function, allowing tumor cells to escape cell death. Examples of tumor suppressor genes commonly mutated in various types of cancers include TP53 (p53), BRCA1, BRCA2, PTEN, APC, and NF1, among others. However, strategies such as REPAIR, which can correct such mutations in tumor cells, offer potential therapeutic options to restore normal tumor suppressor gene function and enhance cell death. In addition, co-administration of other CRISPR-based approaches targeting driver mutations in oncogenes may be necessary for comprehensive and effective cancer therapy. These advancements in genome editing technologies hold promise for precision cancer therapeutics by targeting specific mutations in tumor suppressor genes and oncogenes when used in combination with REPAIR as co-administered strategies. Moreover, the versatility of this strategy can be extended across multiple types of cancers, making it applicable to a wide range of malignancies.

## 6 Tackling limitations: recent advancements in CRISPR/Cas system

In the subsequent sections, the primary constraints of CRISPR technologies are outlined, along with recent advancements aimed at mitigating or overcoming these limitations. One of the biggest constraints is off-target activity. Minimizing off-target activity *in vivo* for CRISPR/Cas genome editing requires careful consideration of factors such as gRNA design, including length and modifications, unavailability of PAM, Cas nuclease variant choice, delivery method, cellular context, and DNA repair mechanisms. Rigorous validation using techniques like whole-genome sequencing or targeted deep sequencing is essential to accurately assess specificity. By optimizing these factors, CRISPR/Cas genome editing can be made more specific *in vivo*.

### 6.1 Utilization of different Cas variants to mitigate off-target activity

Off-target effects continue to be a significant concern, particularly in complex eukaryotic organisms, especially *in vivo* settings for therapeutic applications (Zischewski et al., 2017; Yang and Chen, 2018). Promising strategies for reducing off-target activity (also discussed partially in the above section) in CRISPR/Cas genome editing include the use of improved Cas variants derived from *Streptococcus*. High-fidelity Cas9 variants like Cas9-HF1, eSpCas9, HypaCas9, and Sniper Cas9, demonstrate enhanced specificity and reduced off-target cleavage compared to wild-type Cas9 (Table 2). By optimizing and engineering CRISPR systems, it is possible to minimize off-target effects through strategies such as increasing the cleavage specificity of the nucleases or reducing their functional activity time frame. These advancements in Cas proteins provide potential solutions for enhancing on-target specificity and minimizing off-target effects in gene editing applications.

**TABLE 2:** Cas nuclease variants. Overview and evaluation of off-target effects.

Name	Description	Remarks on off-target activity	Ref.
SpCas9	Most widely used Cas protein for genome editing. Binds a specific guide RNA to generate a double-stranded DNA break (DSB) at the target site	Can exhibit off-target activity, leading to unintended DNA cleavage at off-target sites with similar sequences to the intended target site. Off-target activity can be minimized by careful sgRNA design and Cas9 protein engineering	Heler et al. (2017)
dCas9–FokI	A fusion of deactivated Cas9 (dCas9) and FokI nuclease domain, used for customizable DNA cleavage. Requires two dCas9-FokI complexes to bind to adjacent target sites for cleavage	Off-target activity is dependent on the FokI nuclease domain and can be minimized by optimizing the dCas9-FokI fusion and target site selection	Guilinger et al. (2014)
SpCas9–HFI	A Cas9 variant with increased specificity and reduced off-target activity. Contains mutations in the RuvC1 domain to improve specificity	Shows reduced off-target activity compared to wild-type SpCas9, but careful sgRNA design and experimental optimization are still important to minimize off-target effects	Kleinstiver et al. (2016)
Nickase	A Cas9 variant that cleaves only one strand of DNA, generating a single-stranded break. Requires two nickases to bind to adjacent target sites for DSB formation	Off-target activity is reduced compared to wild-type Cas9 due to the requirement of two nickases for DSB formation, but careful target site selection is still important to minimize off-target effects	Shen et al. (2014)
Sniper–Cas9	A Cas9 variant with increased specificity and reduced off-target activity. Contains mutations in the RuvC2 domain to improve specificity	Shows reduced off-target activity compared to wild-type Cas9, but further studies are needed to fully understand its specificity and potential off-target effects	Slaymaker et al. (2016)
eSpCas9	An enhanced version of SpCas9 with reduced off-target activity. Contains mutations in both the REC3 and REC2 domains to improve specificity	Exhibits reduced off-target activity compared to wild-type SpCas9, but careful sgRNA design and experimental optimization are still important to minimize off-target effects	Okafor et al. (2019)
evoCas9	An evolved version of SpCas9 with increased specificity and reduced off-target activity. Contains multiple mutations to improve specificity	Shows improved specificity and reduced off-target activity compared to wild-type SpCas9, but further studies are needed to fully understand its specificity and potential off-target effects	Casini et al. (2018)
xCas9	A smaller Cas9 variant with increased specificity and reduced off-target activity. Contains mutations in the REC3 domain to improve specificity	Exhibits reduced off-target activity compared to wild-type SpCas9, but careful sgRNA design and experimental optimization are still important to minimize off-target effects	Hu et al. (2018)
VRER–SpCas9	A Cas9 variant with increased specificity and reduced off-target activity. Contains mutations in the REC1 domain to improve specificity	Shows reduced off-target activity compared to wild-type SpCas9, but further studies are needed to fully understand its specificity and potential off-target effects	Kleinstiver et al. (2015)
HypaCas9	A hyper-accurate Cas9 variant with enhanced specificity and minimal off-target activity. Contains multiple mutations to improve specificity	Exhibits high specificity with minimal off-target activity compared to wild-type SpCas9, making it a promising option for precise genome editing	Ikeda et al. (2019)

### 6.2 Fine-tuning gRNA for improved off-target control

Innovative strategies have emerged to address the challenge of off-target activity in CRISPR systems. One promising approach involves engineering the guide RNA, which plays a critical role in target recognition. The specificity of guide RNA in CRISPR systems can be significantly influenced by various parameters, including GC content, length, and mismatches. Recent research has shown that optimizing the GC content and length of the gRNA can lead to improved specificity at the target site, thereby mitigating off-target cleavage (Naeem et al., 2020).

The length of the gRNA can impact its specificity in several ways. A shorter gRNA may have reduced specificity as it may have more potential off-target sites with partial complementarity. On the other hand, a longer gRNA may have improved specificity but may also have reduced efficiency in guiding Cas9 to the target site due to steric hindrance or reduced stability. Recent studies have shown that optimizing the length of the gRNA can help achieve optimal specificity. For example, gRNAs shorter than 20 nt, also known as truncated gRNAs, have been used to reduce off-target effects (Fu et al., 2014). Truncated gRNAs with lengths ranging from 17 to 19 nt have been shown to exhibit improved specificity while maintaining reasonable efficiency in target cleavage. Moreover, chemical modifications, such as 2′-O-methyl or phosphorothioate modifications, have been explored to enhance gRNA stability and minimize off-target activity (Ryan et al., 2018). Furthermore, in addition to the length of the guide RNA, mismatches between the guide RNA and target DNA also influence the specificity of CRISPR/Cas9 (Lin et al., 2014). Investigators have demonstrated that the tolerance of SpCas9 to mismatches between the guide RNA and target DNA is contingent upon the specific sequence context. Notably, the number, position, and distribution of mismatches play a crucial role in determining the sensitivity of SpCas9 to these variations (Hsu et al., 2013).

Besides the engineering of the gRNA, several methods have been developed for detecting and tackling off-target activity in CRISPR systems. Multiple experimental cell-based approaches such as DISCOVER-seq (Wienert et al., 2020), breaks labeling *in situ* and sequencing (BLISS) (Yan et al., 2017), integrase-defective lentiviral vector (IDLV)-mediated DNA break capture (Wang X. et al., 2015), linear amplification-mediated high-throughput genome-wide sequencing (LAM-HTGTS) (Hu et al., 2016) as well as *in vitro* methods like Digenome-Seq (Park et al., 2017), SITE-seq (Cameron et al., 2017), and circularization for *in vitro* reporting of cleavage effects by sequencing (CIRCLE-seq) (Tsai et al., 2017) allow for precise detection of DSB sites and off-target cleavage identification. Similarly, computational systems such as the Cas-OFFinder algorithm (Bae et al., 2014), the CFD scoring system (Doench et al., 2016) and the machine learning-based approach Elevation (Listgarten et al., 2018) have been instrumental in predicting off-target activity in CRISPR systems.

### 6.3 Anti-CRISPR proteins

Anti-CRISPR proteins, which are naturally occurring proteins that can inhibit the activity of Cas nucleases, have emerged as a promising strategy to mitigate off-target effects in CRISPR gene editing. These proteins can bind to Cas nucleases and prevent their binding to target DNA, thereby reducing the risk of off-target cleavage. Several types of anti-CRISPR proteins have been identified and characterized, including AcrIIA and AcrIIC subfamilies, which have been shown to bind specifically to different Cas nuclease domains, such as the RuvC, HNH, and RuvC-like domains (Harrington et al., 2017; Knott et al., 2019). These interactions can effectively inhibit the activity of Cas nucleases, providing a means to modulate their activity and improve the specificity of CRISPR gene editing. Studies have demonstrated that the expression of anti-CRISPR proteins in human cells can significantly reduce the frequency of off-target mutations induced by Cas9, Cas12a, and Cas13 nucleases (Shin et al., 2017; Watters et al., 2018; Meeske et al., 2020). Additionally, anti-CRISPR proteins have been used in combination with CRISPR gene editing to achieve precise and specific genome modifications, such as gene insertion and deletion, without inducing off-target mutations (Pawluk et al., 2016; Rauch et al., 2017). Further research in this area is warranted to optimize the use of anti-CRISPR proteins in different applications and ensure their safety and efficacy in clinical settings.

### 6.4 Precision genome editing techniques: base editing and prime editing to mitigate off-target effects

Base editing is a precise genome editing technique that allows for targeted modification of specific nucleotide bases in DNA without creating double-strand breaks. It involves the use of a modified Cas nuclease, such as Cas9 nickase or Cas13, fused with a catalytically inactive deaminase enzyme. This deaminase enzyme can convert one base to another at the target site (e.g., C to T or A to G). One of the advantages of base editing is its high precision, as it does not rely on DNA repair mechanisms that may introduce off-target mutations. Additionally, base editing has the potential to reduce off-target activity as it depends on the target site being accessible to the Cas nuclease and deaminase, and the deaminase enzyme has its own specificity for the target base change.

Prime editing is another precise genome editing technique that offers increased specificity compared to traditional CRISPR/Cas systems. It involves the use of a catalytically impaired Cas9 nuclease fused with a reverse transcriptase enzyme, along with a prime editing guide RNA (pegRNA) that contains both a guide sequence for target DNA binding and a template for DNA synthesis (Anzalone et al., 2019). This allows for precise insertion, deletion, or substitution of specific DNA sequences at the target site. Prime editing offers increased specificity as it avoids the formation of double-stranded breaks, and the editing event is guided by both the pegRNA and the template, reducing the reliance on DNA repair mechanisms that can introduce off-target mutations.

Despite the advantageous properties of base and prime editors, previous studies have reported their limitations concerning off-target effects (Slesarenko et al., 2022). However, recent articles have introduced cell-based systems (Kwon J. et al., 2022) and deep learning *in silico* models (Yu et al., 2023), as previously described in section 2.2, to mitigate the off-target activity of these genome editing techniques. The applicability of these approaches for therapeutic use to decrease off-target activity needs thorough validation *in vivo* to ensure their efficacy and safety in complex biological systems.

### 6.5 Delivery

The success of *in vivo* CRISPR/Cas therapy hinges on the development of efficient and safe delivery systems capable of precisely and selectively delivering the CRISPR components into target tissues. Viral vectors, gold nanoparticles, and polymer/lipid-based nanoparticles have emerged as promising delivery systems, each with its own advantages and limitations.

Viral vectors, particularly adeno-associated virus (AAV) vectors, have emerged as a leading delivery system for *in vivo* CRISPR/Cas gene editing. AAV vectors possess high infectivity, enable long-term transgene expression, and allow tissue-specific targeting, rendering them widely used in preclinical and clinical studies. Selecting specific AAV serotypes with distinct tropism has enabled efficient targeting of various organs, such as the liver, lungs, and central nervous system. However, challenges related to immune responses, off-target effects, and limited cargo capacity persist, requiring ongoing improvements for optimal use in clinical applications. Several ongoing attempts have been made to reduce cargo size. One such example is Prophylactic Antiviral CRISPR in human cells (PAC-MAN), which was developed utilizing the VI-D CRISPR/Cas13d variant derived from *Ruminococcus flavefaciens* (Abbott et al., 2020). This particular variant was selected due to its compact size, facilitating efficient packaging in viral vehicles, as well as its remarkable specificity and robust catalytic activity within human cells.

Chemical delivery methods offer alternative strategies for *in vivo* CRISPR/Cas gene editing. Gold nanoparticles have gained attention as potential carriers due to their unique physical and chemical properties, facilitating efficient cellular uptake and delivery of CRISPR components. Successful applications of gold nanoparticles have been reported in various cell types, including stem cells and cancer cells (Lee et al., 2017; Wang et al., 2018). However, addressing challenges related to toxicity and limited cargo capacity is crucial to fully exploit their potential in clinical settings.

Polymer/lipid-based nanoparticles are another promising class of chemical delivery systems for *in vivo* CRISPR/Cas gene editing. These nanoparticles offer advantages such as high loading capacity, stability, and tunable surface properties. Encapsulating the CRISPR components within these nanoparticles enables efficient cellular uptake and protection against degradation. Ongoing research focuses on enhancing delivery efficiency and targeting specificity of polymer/lipid-based nanoparticles to overcome current limitations, including off-target effects and immunogenicity.

A compromise between viral and nonviral delivery platforms is provided by extracellular vesicles (Evs) and virus-like particles (VLPs). Evs are vesicular bodies of cell origin that resemble viruses lacking genomes. If the viral envelope and viral structural proteins are assembled into Evs, the term virus-like particle (VLP) is used. Compared with other approaches, extracellular vesicles and virus-like particles represent a safe, efficient, and transient strategy for CRISPR/Cas delivery. Recently, systems based on extracellular vesicles were reported to deliver Cas9 both *in vitro* and *in vivo* (Montagna et al., 2018; Campbell et al., 2019; Mangeot et al., 2019; Gee et al., 2020). The potential and limitations of EV-based approaches have been extensively described elsewhere (Yip, 2020).

Despite the progress made in developing delivery systems for CRISPR/Cas gene editing, several challenges remain. These include off-target effects, immune responses, limited cargo capacity, and inefficient delivery to certain cell types or tissues. To address these limitations, researchers are exploring strategies such as modifying viral capsids, engineering nanoparticles for improved stability and targeting, and employing combinatorial approaches involving multiple delivery systems. The continued advancement of delivery technologies will undoubtedly contribute to the broader adoption of CRISPR/Cas gene editing, opening up new possibilities for therapeutic interventions and precision medicine.

### 6.6 Addressing immunotoxicity concerns and repeated administration challenges

Gene therapy-related clinical trials have shown that transgene expression from an AAV vector can persist for up to 10 years in humans. Similarly, one of the challenges in CRISPR gene editing is the prolonged expression of Cas9 and guide RNA *in vivo*, which can potentially trigger immunotoxicity and off-target editing. Studies have shown that a significant portion of the human population possesses pre-existing anti-Cas9 antibodies against commonly used bacterial orthologs, such as SaCas9 and SpCas9 (Charlesworth et al., 2019). Additionally, adeno-associated virus (AAV) vectors, which are commonly used to deliver CRISPR components for gene therapy, can trigger immune responses due to the presence of anti-capsid antibodies that neutralize AAV, making repeated administration less effective.

To address these concerns, researchers have explored different Cas9 orthologs and AAV serotypes based on sequence similarities and predicted binding strength to major histocompatibility complex (MHC) class I and class II molecules. While no ortholog or serotype has been found to completely evade immune recognition, Moreno *et al.* identified three Cas9 orthologs, namely, SpCas9, SaCas9, and *Campylobacter jejuni* Cas9 (CjCas9), that showed robust editing efficiency and tolerated repeated administration with reduced immunogenic toxicity in mice immunized against AAV and Cas9 (Moreno et al., 2019). However, pre-existing immunity against SpCas9 and SaCas9 in humans leaves CjCas9 as the only current option for repeated gene therapy in this cohort of patients. Nevertheless, further investigation is needed to establish the safety and efficacy of CjCas9 for clinical use, as it has not been well-studied compared to the other two orthologs. Additionally, the expression of Cas9 in mouse models has been shown to elevate the frequencies of immune cells and trigger cellular immune responses, highlighting the need for careful consideration of immunotoxicity in CRISPR-based therapeutic approaches, particularly in the context of repeated administration and pre-existing immunity against the carrier or Cas9.

## 7 Discussion

The CRISPR/Cas system has revolutionized the field of genome editing, offering unprecedented opportunities for the development of novel therapeutic strategies. In this review, we have explored the mechanisms of CRISPR/Cas9 genome editing and its expansion through the CRISPR/Cas toolkit.

In the context of cancer therapy, the *in vivo* applications of CRISPR/Cas have shown significant promise. The ability to target patient-specific mutations using CRISPR/Cas9 offers a personalized approach to treatment. By precisely editing the cancer genome, it becomes possible to disrupt oncogenic drivers or restore tumor suppressor functions, thereby potentially inhibiting cancer progression. Additionally, the non-patient-specific universal targeting approach allows for the development of off-the-shelf therapies, providing a more accessible and cost-effective solution for treating a wide range of cancers. Yet, the high variability within the mutational landscape of tumor cells remains a major challenge.

While the potential of CRISPR/Cas therapy in cancer treatment is undeniable, there are still several opportunities and challenges that need to be addressed. One promising avenue is the combination of CRISPR/Cas with other cancer therapies to enhance efficacy. By synergizing CRISPR/Cas with existing treatment modalities such as chemotherapy or immunotherapy, it may be possible to achieve a more comprehensive and effective approach to cancer therapy. Multiplexing CRISPR/Cas for multi-targeted regulation also holds great potential for tackling the heterogeneity and complexity of cancer genomes.

One of the challenges faced by CRISPR/Cas in cancer therapy is the presence of protospacer adjacent motif (PAM) limitations. However, recent advancements have shown promise in overcoming this drawback. Co-targeting oncogenes and tumor suppressors using multiplexing CRISPR/Cas can circumvent the PAM constraints and enable simultaneous targeting of multiple cancer-related genes. This approach has the potential to enhance the precision and efficacy of CRISPR/Cas therapy.

Moreover, several strategies have been developed to mitigate off-target effects associated with CRISPR/Cas. The utilization of different Cas variants, such as Cas12a or Cas13, can help mitigate off-target activity due to their unique target recognition properties. Fine-tuning guide RNA design and optimization can also improve off-target control by reducing unintended binding events. Furthermore, emerging precision genome editing techniques such as base editing and prime editing offer additional tools to mitigate off-target effects and increase the specificity of CRISPR/Cas-mediated genome modifications.

Another challenge for the successful employment of the CRISPR/Cas technology, especially for *in vivo* applications, is the development of safe and efficient delivery systems. Depending on the specific purpose, multiple delivery platforms including viral systems, gold nanoparticles, and lipid-based nanoparticles are suitable options for efficient editing. Considering their versatility and non-integrating properties, in particular extracellular vesicles and virus-like particles have emerged as promising strategies at the *in vivo* level. Further optimization to tackle primarily technical limitations will certainly encourage the use of CRISPR/Cas gene editing for precision medicine. Along with the development of appropriate delivery systems, the selection of suitable Cas orthologs may allow circumventing immunotoxicity concerns and administration issues.

In conclusion, the field of CRISPR/Cas-mediated therapy in cancer is rapidly advancing, with significant potential for personalized and precise treatment strategies. The ability to target patient-specific mutations, multiplex gene regulation, and overcome limitations associated with off-target effects are key areas of progress. However, further research and development are required to optimize the safety, delivery, and scalability of CRISPR/Cas therapies for clinical translation. The utilization of CRISPR/Cas9 technology in an *ex vivo* manner has the potential for immediate therapeutic benefits for certain diseases. However, to achieve a more comprehensive and impactful clinical outcome, the direct administration of CRISPR/Cas9 to patients is required. This approach would enable a more vigorous application of the technology and more significant advancement in the treatment of various medical conditions. With continued advancements and refinement, CRISPR/Cas holds immense promise for transforming the landscape of cancer therapy and improving patient outcomes.
